# Functional and Structural Neural Network Characterization of Serotonin Transporter Knockout Rats

**DOI:** 10.1371/journal.pone.0057780

**Published:** 2013-02-25

**Authors:** Kajo van der Marel, Judith R. Homberg, Willem M. Otte, Rick M. Dijkhuizen

**Affiliations:** 1 Biomedical MR Imaging and Spectroscopy Group, Image Sciences Institute, University Medical Center Utrecht, Utrecht, The Netherlands; 2 Department of Cognitive Neuroscience, Centre for Neuroscience, Donders Institute for Brain, Cognition, and Behaviour, Radboud University Nijmegen Medical Centre, Nijmegen, The Netherlands; 3 Department of Pediatric Neurology, Rudolf Magnus Institute of Neuroscience, University Medical Center Utrecht, Utrecht, The Netherlands; University of New Mexico, United States Of America

## Abstract

Brain serotonin homeostasis is crucially maintained by the serotonin transporter (5-HTT), and its down-regulation has been linked to increased vulnerability for anxiety- and depression-related behavior. Studies in 5-HTT knockout (5-HTT^-/-^) rodents have associated inherited reduced functional expression of 5-HTT with increased sensitivity to adverse as well as rewarding environmental stimuli, and in particular cocaine hyperresponsivity. 5-HTT down-regulation may affect normal neuronal wiring of implicated corticolimbic cerebral structures. To further our understanding of its contribution to potential alterations in basal functional and structural properties of neural network configurations, we applied resting-state functional MRI (fMRI), pharmacological MRI of cocaine-induced activation, and diffusion tensor imaging (DTI) in 5-HTT^-/-^ rats and wild-type controls (5-HTT^+/+^). We found that baseline functional connectivity values and cocaine-induced neural activity within the corticolimbic network was not significantly altered in 5-HTT^-/-^ versus 5-HTT^+/+^ rats. Similarly, DTI revealed mostly intact white matter structural integrity, except for a reduced fractional anisotropy in the genu of the corpus callosum of 5-HTT^-/-^ rats. At the macroscopic level, analyses of complex graphs constructed from either functional connectivity values or structural DTI-based tractography results revealed that key properties of brain network organization were essentially similar between 5-HTT^+/+^ and 5-HTT^-/-^ rats. The individual tests for differences between 5-HTT^+/+^ and 5-HTT^-/-^ rats were capable of detecting significant effects ranging from 5.8% (fractional anisotropy) to 26.1% (pharmacological MRI) and 29.3% (functional connectivity). Tentatively, lower fractional anisotropy in the genu of the corpus callosum could indicate a reduced capacity for information integration across hemispheres in 5-HTT^-/-^ rats. Overall, the comparison of 5-HTT^-/-^ and wild-type rats suggests mostly limited effects of 5-HTT genotype on MRI-based measures of brain morphology and function.

## Introduction

Serotonin (5-HT) is an ancient neurotransmitter that subserves many brain functions, including emotional processing and cognitive flexibility [1], [2]. As the serotonin transporter (5-HTT) is a critical modulator of serotonin homeostasis, its dysfunction has been linked to a variety of neuropsychiatric disorders, including anxiety and depression, and substance abuse [3]. A well-known common polymorphism in the promoter region of 5-HTT (5-HTTLPR; 5-HTT-linked polymorphic region) has been studied intensively with regard to anxiety-related traits and vulnerability to depression [4]. More specifically, individuals carrying the low activity (short; s) allelic variant of the 5-HTTLPR show anxiety-like traits and exhibit an increased risk to depression upon stress exposure in early life [5], [6]. As these phenotypes have been noted in 5-HTT knockout (5-HTT^-/-^) rodents as well [7], there is strong believe that inherited 5-HTT down-regulation is associated with increased sensitivity to adverse environmental stimuli [8]. Whereas 5-HTT studies are consequently strongly biased to stress effects in 5-HTT genetically compromised individuals, there is also evidence that 5-HTT genotype affects the sensitivity to rewarding environmental stimuli, like drugs of abuse [9]. This was particularly evident in previous studies showing that 5-HTT^-/-^ mice and rats display increased cocaine-induced locomotor activity, cocaine-induced conditioned place preference, and intravenous cocaine self-administration [9]–[13]. Cocaine is a psychomotor stimulant that acts through inhibition of the serotonin, dopamine and norepinephrine transporter [14]. Whereas the absence of 5-HTT, and thus a failure to increase 5-HT release (JR Homberg, JD Olivier, unpublished observations), may explain the hyperresponsivity to cocaine in 5-HTT^-/-^ rats, there is no clear evidence that such a pharmacological process explains the behavioral response in 5-HTT^-/-^ rats [9]. Alternatively, cocaine may increase mood states [15]. Thereby its intake may serve to alleviate the negative emotional state experienced by 5-HTT^-/-^ rats [16]. As we hypothesized previously, it is possible that developmental increases in 5-HT levels due to reduced 5-HTT availability lead to alterations in the wiring of corticolimbic structures implicated in anxiety-like traits, which are targeted by rewarding environmental stimuli, like cocaine [8]. Structural and functional magnetic resonance imaging (MRI) studies in humans have revealed that 5-HTTLPR s-allele carriers exhibit reduced perigenual anterior cingulate cortex (pACC) and amygdala volumes, and reduced connectivity between the two [17]. Since the pACC controls emotional responses mediated by the amygdala, it is thought that this decreased cortical control over emotion contributes to the anxiety-like traits in s-allele carriers. This is further corroborated by the observation that blood oxygenation level-dependent (BOLD) responses are increased in the amygdala upon exposure to pictures of fearful faces in functional MRI (fMRI) studies [18]. In 5-HTT^-/-^ rodents, immunohistochemical studies have revealed changes in the dendritic branching and number of spines in amygdala and prefrontal pyramidal neurons [19], [20], which may correspond to the structural and functional changes noted in s-allele carriers.

MRI can also be employed in small animal models, which provides a unique opportunity to link rodent and human data. Recent findings from manganese-enhanced MRI of the 5-HTT^-/-^ mouse suggest that there are alterations in reward pathways connecting prefrontal cortex, nucleus accumbens, caudate-putamen, and thalamic nuclei [21]. However, it remains unclear to what extent compromised 5-HTT function in rodents may lead to alterations in basal functional and structural properties of neural network configurations, and whether these properties contribute to cocaine supersensitivity and heightened stress sensitivity in 5-HTT^-/-^ animals. To obtain a comprehensive view of the differences in structural and functional network configurations in 5-HTT^-/-^ rats and wild-type (5-HTT^+/+^) controls, we here applied, for the first time, various MRI paradigms. Brain function in baseline ‘resting’ condition, and upon cocaine-stimulation, was assessed with resting-state fMRI, and a pharmacological MRI paradigm, respectively. Measures of underlying structural integrity were obtained from Diffusion Tensor Imaging (DTI) using a high-angular resolution diffusion imaging (HARDI) scheme [22]. Finally, we applied various seed-based and voxel-based analyses to explore differences in both local and global functional and structural properties of neural networks between 5-HTT^+/+^ and 5-HTT^-/-^ rats.

## Materials and Methods

### Ethics statement

All experiments were approved by the Committee for Animal Experiments of the University Medical Center Utrecht, The Netherlands (2007.I.07.098), and all efforts were made to minimize animal suffering and to reduce the number of animals used.

### Animals

A total of 31 adult male Wistar rats was used in the study, of which 13 5-HTT^+/+^ (body weight 557.8 ± 76.5 g) and 18 5-HTT^-/-^ (Slc6a4^1Hubr^; body weight 492.6 ± 24.2 g). Experimental animals were derived from crossing heterozygous 5-HTT knockout rats that were outcrossed for at least ten generations, with wild-type Wistar rats obtained from Harlan Laboratories (The Netherlands). After weaning at the age of 21 days, ear cuts were taken for genotyping, which was performed by Kbiosciences (Hoddesdon, United Kingdom). A 12-h light–dark cycle was maintained, with lights on at 08.00.

Animals were anesthetized with 5% isoflurane (2–2.5% maintenance) in air/O_2_ (2:1). Two tail veins were cannulated for administration of pancuronium bromide (2 mg/ml) and cocaine HCl dissolved in saline (1 mg/ml), respectively. The left femoral artery was cannulated for blood pressure measurements and blood gas analysis. Three 5-HTT^-/-^ animals were excluded because of complications during surgical preparation. One 5-HTT^+/+^ animal was excluded from the pharmacological MRI analysis, because of an obstructed intravenous cannula, and because of mechanical ventilation failure two animals (one 5-HTT^+/+^ and one 5-HTT^-/-^) were excluded from the DTI analysis.

### MRI acquisition

MRI measurements were conducted on a 4.7T horizontal bore MR system (Varian, Palo Alto, CA), with use of a Helmholtz volume coil (90 mm diameter) and an inductively coupled surface coil (25 mm diameter) for signal excitation and detection, respectively. Rats were mechanically ventilated (CIV 101, Columbus Instruments, Columbus, OH) with 1% end-tidal isoflurane in air/O_2_ (2:1) and received a continuous infusion of 1 mg/kg/h pancuronium bromide to prevent movement. During MRI, blood oxygen saturation and heart rate were monitored using a pulse oximeter (8600V, Nonin Medical, Plymouth, MN), end-tidal CO_2_ with a capnograph (Multinex 4200, Datascope Corporation, Paramus, NJ), and mean arterial blood pressure (MABP) from a femoral artery cannulation with a small bore polyethylene tubing connected to a pressure transducer (SA Instruments, Stony Brook, NY). Body temperature was maintained at 37.0 ± 0.5°C. Before and after resting-state fMRI and pharmacological MRI, arterial CO_2_ tension (P_a_CO_2_) was measured from a sample of 0.15 ml arterial blood (i-STAT, Abbott Laboratories, Princeton, NJ).

First, 10 minutes of resting-state fMRI with high temporal resolution was performed using a T_2_*-weighted single-shot gradient echo EPI sequence (repetition time (TR)/echo time (TE) = 500/19 ms; 35° flip angle; 64×64 matrix; 0.5×0.5 mm^2^ voxels; 7×1.5 mm coronal slices; 1200 BOLD images). Subsequently, gradient-echo multi-slice T_2_*-weighted pharmacological MRI was performed (TR/TE = 468.75/35 ms; 60° flip angle; 64×64 matrix; 0.5×0.5 mm^2^ voxels; 12×1.0 mm coronal slices) with 10 minutes (20 images) of baseline measurements followed by 40 minutes (80 images) acquisition after injection of 1 mg/kg cocaine. Structural connectivity was quantified from a HARDI acquisition scheme that was optimized for whole brain tractography, and also included parameter maps derived from the diffusion tensor, i.e. mean diffusivity and fractional anisotropy. A diffusion-weighted four shot spin-echo EPI sequence (TR/TE = 3500/26 ms; 64×64 matrix; 0.5×0.5 mm^2^ voxels; 25×0.5 mm transversal slices; *b* = 1250 s/mm^2^, δ = 6 ms, Δ = 11 ms; 50 diffusion-weighted images in non-collinear directions, and 2 images without diffusion-weighting (*b* = 0)). Anatomical images for registration purposes were obtained with a 3D gradient-echo sequence (TR/TE = 6/2.576 ms; 40° flip angle; 256×128×128 matrix; field of view = 60×40×40 mm^3^; 4 averages).

### Image analysis

Using *ANTS* [23], within-subject functional, diffusion-weighted and anatomical images were non-rigidly aligned, and subsequently registered to an anatomical reference image that was matched to a 3D model of a rat brain atlas [24]. All diffusion-weighted, resting-state fMRI, and pharmacological MRI images were corrected for subject motion using *FLIRT* [25]. Resting-state fMRI and pharmacological MRI images were spatially smoothed (Gaussian kernel, full width at half maximum  =  1.0 mm), and corrected for linear drift.

After spatial smoothing and intensity scaling, non-neuronal contributions to the resting-state fMRI signal were removed by linear regression, as outlined in [26], with (1) the global mean time-varying signal; (2) six motion correction parameter estimates; (3) a linear trend. Low-frequency fluctuations of the resting-state BOLD signal were obtained by band-pass filtering between 0.01 and 0.1 Hz.

Analysis of DTI data was performed using *Diffusion Toolkit* [27]. Briefly, data sets were resampled on an isotropic 0.25 mm^3^ grid, and parameter maps based on voxel-wise estimates of the diffusion tensor were calculated, including fractional anisotropy and mean diffusivity. Whole-brain tractography was obtained from seeds in each voxel according to fractional anisotropy > 0.2 using the interpolated streamline algorithm implementation, with a 35° angle threshold and originating from 10 random seeds in each voxel. Structural connections between any pair of gray matter regions of interest (ROIs; see below) were obtained from tracts that had end points in both ROIs, with a minimum length of 1.0 mm to prevent direct gray matter connections between neighboring areas.

### Regions of interest

Bilateral gray matter ROIs that are mainly within the limbic system and receive serotonergic innervations, were projected from the atlas onto the functional time series for seed-based functional connectivity and pharmacological MRI analysis. ROIs included dorsal and ventral medial prefrontal cortices (PFC (dm) and PFC (vm), respectively) [28], [29], insula (Ins), frontal cortex (FC), ventral and dorsal orbitofrontal cortices (OFC (v) and OFC (d), respectively), nucleus accumbens (NAc), caudate-putamen (CPu), globus pallidus (GP), basal amygdaloid nuclei (Amy), thalamic nuclei (Tha), dorsal and ventral hippocampus (Hip (d) and Hip (v), respectively), substantia nigra (SN), dorsal raphe nuclei (DRN), ventral tegmental area (VTA), and several cortical areas, i.e. visual (ViC), auditory (AuC), temporal (TeC), parietal (PtC), motor (MC), somatosensory (SmC), and retrosplenial cortex (RsC).

Because of the limited field of view and spatial resolution of the resting-state fMRI data, ROIs that were not reliably identified (i.e., spanning less than two voxels) in at least ten subjects per group, i.e. ventral tegmental area, dorsal raphe nuclei, ventral orbitofrontal cortex, and frontal cortex, were excluded from the resting-state fMRI analyses.

For the analysis of white matter structural integrity from DTI parameter maps, white matter ROIs were first outlined on the mean T_2_-weighted *b* = 0 maps. ROIs included the genu and body of the corpus callosum, the anterior part of the anterior commissure, and the anterior part of the internal capsule. An observer - blinded for the animal’s genotype - adjusted individual ROIs using each subject’s *b* = 0 and fractional anisotropy maps.

### Statistical analysis of resting-state fMRI

First, a seed-based analysis of functional connectivity was performed. Functional connectivity was measured as the Fisher *z’*-transformed correlation coefficient *r* between pairs of BOLD low-frequency fluctuation signals, i.e. 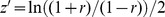. Functional connectivity values of homologous seeds in the left and right hemisphere were averaged to obtain functional connectivity between pairs of inter-hemispheric and intra-hemispheric ROIs. Functional connectivity was either calculated between two seed regions, or between a seed region’s signal and all voxels to obtain functional connectivity maps. One-sample and two-sample *t*-tests determined significant (non-zero) average functional connectivity, and significant differences between groups, respectively. *t*-value maps corresponding to *p* < 0.01 were cluster-corrected (*p* < 0.01, cluster size >160.8 mm^3^ corresponding to *p* < 0.01, as obtained from Monte Carlo simulations implemented in the *AlphaSim* command available in *AFNI* [30], [31]).

Second, to obtain a comprehensive, quantitative view of the complex interactions among brain regions in terms of their functional couplings, we applied a graph theory perspective to analyze global properties of complex functional brain networks [32]–[35]. For each subject, a weighted undirected graph *G  =  (V, W)* was constructed (*R* software; *igraph* package [36]), with a collection of nodes *V* and a collection of edge weights *W*. A node represented a ROI with an associated mean time-varying signal. Edges between each pair of nodes were weighted by the correlation coefficient between their signals. Whereas analysis of binary graphs requires an arbitrary threshold to separate strong from weak edge connections, we computed measures that utilize all edges in a weighted manner, except for edges that have negative weights or represent self-connections [34]. From these graphs we calculated global measures of (1) segregated/modular information processing, i.e. the clustering coefficient (average of local clustering coefficients) [37]; (2) integrated/distributed information processing, i.e. the characteristic path length (average of all shortest path lengths) [38]; and (3) the balance between global and local efficiency of parallel information processing, i.e., ‘small-worldness’ [39], [40]. Clustering coefficients and characteristic path lengths were normalized based on 1000 instances of surrogate random networks obtained through random edge weights permutations for each subject’s graph [41]. When appropriate, effect sizes (Cohen’s *d*) and corresponding *t*-values are reported.

### Statistical analysis of pharmacological MRI

The pharmacological MRI data was analyzed in three different ways: (1) to test whether the BOLD time course after i.v. cocaine injection was different between 5-HTT^+/+^ and 5-HTT^-/-^ animals on a seed-by-seed basis; (2) to assess differences in the peak activation and total activation following cocaine, using a seed-by-seed parametric fit; and (3) to obtain voxel-level maps of group-level neural activity, and differences in the BOLD response, upon cocaine stimulation.

First, we tested on a seed-by-seed basis whether the BOLD response after i.v. cocaine injection was different between the two groups. For each ROI, we tested the mean preprocessed time series data between the groups in a generalized least squares fit (*R* software; *nlme* package [42]). To account for autocorrelation in the BOLD measurements we included a continuous-time first-order autoregressive error term AR(1). Group and time were taken as main effects, and as covariates the P_a_CO_2_ before pharmacological MRI, and the MABP before, and at peak response (between 5 and 15 minutes) after cocaine injection, i.e. %BOLD ∼ group + time + P_a_CO_2 *before*_ + MABP *_before_* + MABP *_peak_*. Means and standard errors of the group effect were estimated using normalized response and predictor variables.

Second, we fitted a three-parameter function *f* dependent on time *t* after cocaine injection, from which we could easily calculate total activation (i.e., area under the curve), and peak activation. Here,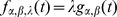 with shape (*α*) and rate (*β*) parameters of a gamma-variate function,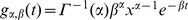, multiplied by a scale factor (*λ*). Fitting was performed in a Bayesian framework using Markov Chain Monte Carlo (MCMC) methods as implemented in *JAGS* [43] and interfaced from *R* (*R2jags* package), to obtain for each subject and ROI a parametric estimate of the signal change relative to the baseline value. In a hierarchical design, group-level distributions structured the between-subject variation in model parameters. Such hierarchical models provide constraints to the fits of individual time courses, thus improving the parameter estimates at the individual ROI level in the presence of noise, and are easily specified in a Bayesian setting. A caveat of this design is that suitable priors need to be specified to obtain robust fits. We have therefore defined prior distribution parameters based on model parameters of initial subject-level fits. From the subject-level model parameter estimates we calculated maximum signal change, and total activation (area under curve). To test for an overall group difference, a linear mixed-model analysis was performed (*R* software; *nlme* package [42]) separately for maximum signal change and area under the curve, taking group and time as fixed main effects, P_a_CO_2 *before*_, MABP *_before_*, and MABP *_peak_* as covariates, and the ROIs per subject as random effects.

Third, we obtained group-level spatial maps of BOLD activation upon cocaine stimulation using a voxel-based generalized least squares fit, with an AR(1) correlation structure to account for autocorrelation in the time series data, for a design matrix that was constructed according to the procedure described in [44]. Briefly, the design matrix consisted of (1) a representative gamma-variate response curve; (2) the frame-by-frame motion correction parameters (six degrees of freedom); (3) the first three components of a singular value decomposition of variations in evolution of the BOLD response as modeled in (1). Each subject was fitted separately, and the estimates of the coefficients of the response function (1) were then analyzed for group differences using a linear model fit, with as covariates the P_a_CO_2 *before*_, and the pre- and post-injection MABP levels, i.e. MABP *_before_* and MABP *_peak_*. *t*-value maps corresponding to *p* < 0.05 were cluster-corrected (cluster size >12.0 mm^3^ corresponding to *p* < 0.05, as obtained from Monte Carlo simulations implemented in the *AlphaSim* command available in AFNI [30], [31]).

In all generalized least squares, linear model, and linear mixed-model fits, missing covariate values were replaced by their group mean values [45].

### Statistical analysis of DTI

First, in a tract-based spatial statistics (TBSS) approach as implemented in *FSL* [46], voxel-wise analysis of white matter structural integrity was performed after projecting fractional anisotropy values onto a common white matter skeleton, to account for residual alignment inaccuracies after non-rigid inter-subject registration and to limited the number of tests.

Second, a seed-based analysis of differences in white matter fractional anisotropy values was performed for the four manually outlined ROIs. Gray matter differences in fractional anisotropy were tested for the ROIs projected from the stereotaxic atlas.

Third, analogous to the complex functional brain networks constructed from the resting-state fMRI functional connectivity values, we constructed from the DTI-based fiber tracts for each subject a complex graph expressing structural connectivity among the 23 bilaterally positioned cortical and subcortical gray matter ROIs. The edges between the regions were weighted by the mean fractional anisotropy along the connecting tracts. As described above, we calculated from these graphs the normalized clustering coefficient and normalized characteristic path length, and small-worldness as the ratio between the two former measures.

## Results

### Resting-state functional MRI

**Figure 1:**
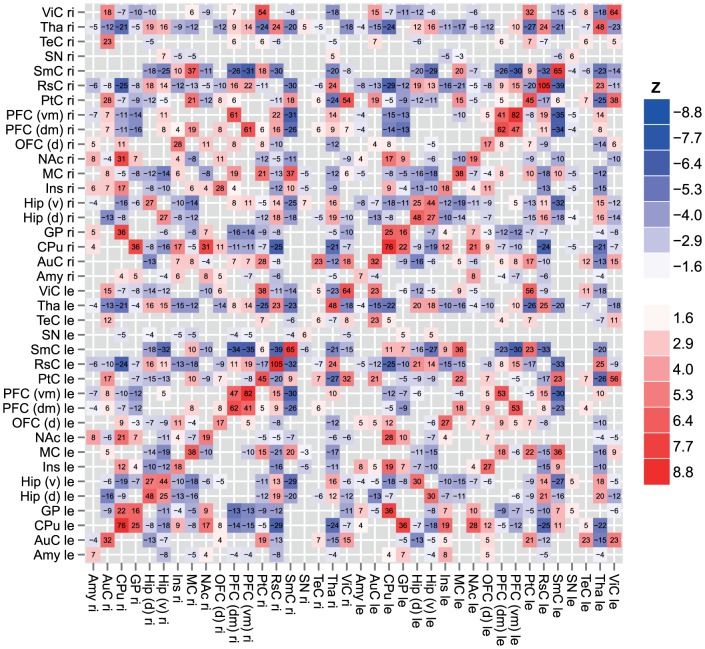
Seed-based resting-state fMRI functional connectivity matrices. Resting-state fMRI-based functional connectivity values were calculated and tested for significance (*p* < 0.05, one-sample *t*-test) across all 5-HTT^+/+^ and 5-HTT^-/-^ animals, among all seed regions in left (le) and right (ri) hemispheres. Significant non-zero functional connectivity values were scaled by 10^2^ and color-coded according to *Z*, the normalized *t*-value. The *p*-values were false discovery rate (FDR) adjusted for multiple comparisons. *Amy*: basal amygdaloid nuclei; *AuC*: auditory cortex; *CPu*: caudate-putamen; *GP*: globus pallidus; *Hip (d)*: dorsal hippocampus; *Hip (v)*: ventral hippocampus; *Ins*: insula; *MC*: motor cortex; *NAc*: nucleus accumbens; *OFC (d)*: dorsal orbitofrontal cortex; *PFC (dm)*: dorsomedial prefrontal cortex; *PFC (vm)*: ventromedial prefrontal cortex; *PtC*: parietal cortex; *RsC*: retrosplenial cortex; *SmC*: somatosensory cortex; *SN*: substantia nigra; *TeC*: temporal cortex; *Tha*: thalamic nuclei; *ViC*: visual cortex.

Figure 1 depicts overall significantly non-zero (false discovery rate (FDR)-adjusted *p* < 0.05, one-sample *t*-test) functional connectivity values among the seed ROIs across all 5-HTT^+/+^ and 5-HTT^-/-^ animals, color-coded with the corresponding *Z*-value. To increase the statistical power of between-group *t*-tests and obtain functional connectivity between pairs of inter-hemispheric and intra-hemispheric ROIs, functional connectivity values of homologous seeds in the left and right hemisphere were averaged. Strong inter-hemispheric functional connectivity between homologous ROIs was observed for almost all seeds. To assess whether group-level functional connectivity values differed between the 5-HTT^+/+^ and 5-HTT^-/-^ groups, we performed pairwise two-sample *t*-tests (Figure 1, bottom row). Differences in functional connectivity between 5-HTT^+/+^ and 5-HTT^-/-^ rats were not significant after FDR-correction for multiple comparisons. Uncorrected *p*-values (*p* < 0.05) show differences between 5-HTT^+/+^ and 5-HTT^-/-^ rats in intra-hemispheric functional connectivity between dorsal hippocampus and auditory cortex (*t*(20)  =  2.4; effect size: Cohen’s *d*  =  1.07), caudate-putamen (*t*(24)  =  -2.1; *d*  =  -0.87) and dorsal orbitofrontal cortex (*t*(13)  =  -3.0; *d*  =  -1.65), between somatosensory cortex and insula (*t*(24)  =  2.2; *d*  =  0.89), and between visual cortex and somatosensory cortex (*t*(18)  =  2.3; *d*  =  1.06) and ventromedial prefrontal cortex (*t*(22)  =  -2.4; *d*  =  -1.02). Inter-hemispheric functional connectivity was significantly different (*p* < 0.05, uncorrected) between dorsomedial prefrontal cortex and amygdala, (*t*(24)  =  -2.1; *d*  =  -0.87); between caudate-putamen and nucleus accumbens (*t*(24)  =  -2.5; *d*  =  -1.02) and substantia nigra (*t*(20)  =  -2.2; *d*  =  -0.99); between temporal cortex and insula (*t*(18)  =  -2.2; *d*  =  -1.03) and retrosplenial cortex (*t*(18)  =  -2.2; *d*  =  -1.05); and between visual cortex and insula (*t*(21)  =  -2.5; *d*  =  -1.07) and ventromedial prefrontal cortex (*t*(22)  =  -2.5; *d*  =  -1.06).

**Figure 2:**
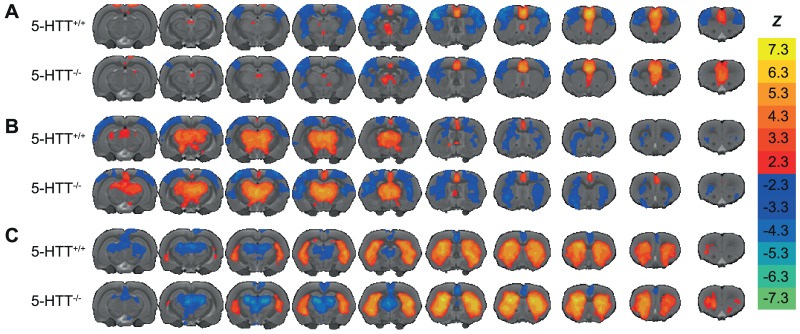
Maps of seed-based resting-state fMRI functional connectivities. The resting-state fMRI signal of a seed region was correlated with all voxels in the brain. For each voxel, a one-sample *t*-test was performed to determine whether the signal at that location correlated significantly with the seed-region. Group-level resting-state fMRI-based functional connectivity maps are displayed for three different seed regions. Voxels that exhibit significant (*p* < 0.01, cluster-corrected) functional connectivity with a seed region in (A) ventromedial prefrontal cortex, (B) thalamic nuclei, and (C) caudate-putamen, are color-coded according to *Z*-value thresholded at 2.3 (*p* < 0.01) for both positive (red to yellow) and negative (blue to light blue) correlations between the filtered time-varying signals, and overlaid on a multi-slice anatomical rat brain template.

Figure 2 shows functional connectivity maps (seed-based, only significant non-zero functional connectivity after cluster-correction) with seeds in ventromedial prefrontal cortex, thalamus, and caudate-putamen, for both groups. Ventromedial prefrontal cortex displayed significant positive functional connectivity with retrosplenial cortex, and thalamic nuclei. On the other hand, thalamic nuclei showed negative functional connectivity values with caudate-putamen, nucleus accumbens, and cortical somatosensory areas.

**Figure 3:**
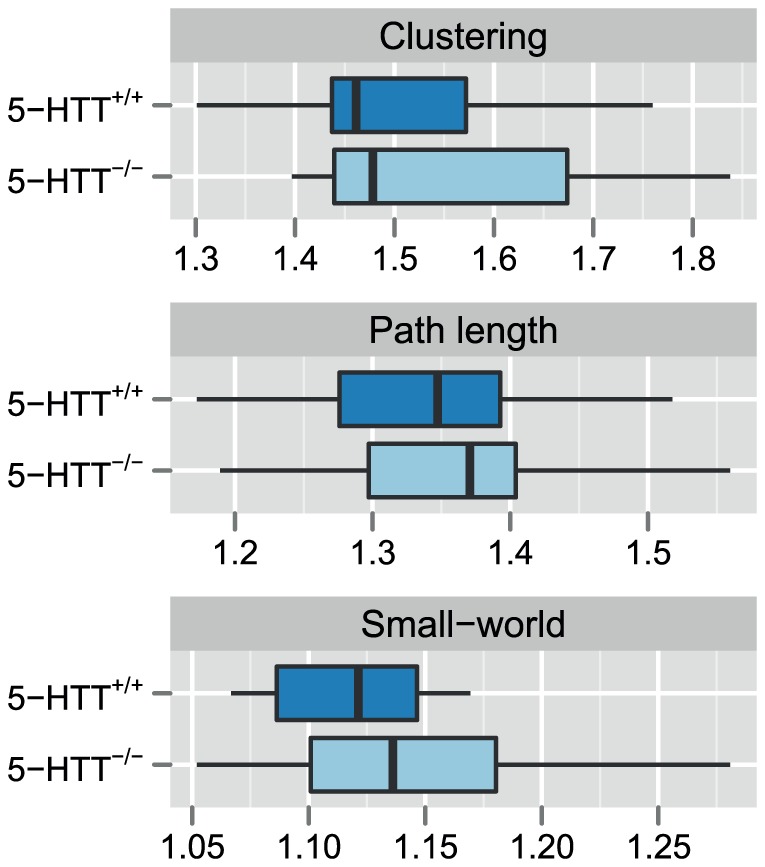
Network parameters from seed-based graph analysis of resting-state fMRI functional connectivity. Functional brain networks were constructed from seed-based resting-state fMRI functional connectivity values. The boxplots display three global network parameters that capture key properties of the functional brain networks, and were calculated on weighted graphs: the clustering coefficient (i.e., average of local clustering coefficients), the characteristic path length (i.e., average of shortest path lengths), and the small-worldness (i.e., ratio of normalized clustering coefficient and normalized characteristic path length).

The functional brain networks constructed from seed-based resting-state fMRI functional connectivity values were characterized by the normalized clustering coefficient, the normalized characteristic path length, and the ratio between these two values, i.e., small-worldness (Figure 3). We found that both characteristic path length and clustering coefficient were larger than observed in random networks, and the networks exhibited ‘small-world’ properties, i.e. the ratio between normalized clustering coefficient and normalized characteristic path length was slightly larger than 1, indicating a more clustered organization while maintaining relatively low shortest path lengths. The normalized clustering coefficient was found to be 1.50 ± 0.12 in 5-HTT^+/+^ and 1.55 ± 0.15 in 5-HTT^-/-^ (*d*  =  -1.50), the normalized characteristic path length 1.34 ± 0.09 in 5-HTT^+/+^ and 1.36 ± 0.10 in 5-HTT^-/-^ (*d*  =  -0.73), and small-worldness 1.12 ± 0.04 in 5-HTT^+/+^ and 1.14 ± 0.06 in 5-HTT^-/-^ (*d*  =  -1.81). We found no significant differences in these global resting-state fMRI functional connectivity graph parameters between wild-types and knockouts.

### Pharmacological MRI

Before MRI, ventilation volume was determined by body weight, and subsequent adjustments were aimed at maintaining normal P_a_CO_2_ levels. To account for within- and between-group variations in baseline P_a_CO_2_, and variations in blood pressure before and after cocaine injection, these values were included as covariates in the statistical analyses. Baseline P_a_CO_2_ was 39.2 ± 2.5 mmHg in 5-HTT^+/+^ and 34.7 ± 7.3 mmHg in 5-HTT^-/-^. MABP went from 118.2 ± 41.8 mmHg (baseline) to 117.1 ± 39.2 mmHg (between 5 and 15 minutes after cocaine injection) in 5-HTT^+/+^, and from 126.8 ± 26.3 mmHg to 129.2 ± 25.7 mmHg in 5-HTT^-/-^.

**Figure 4:**
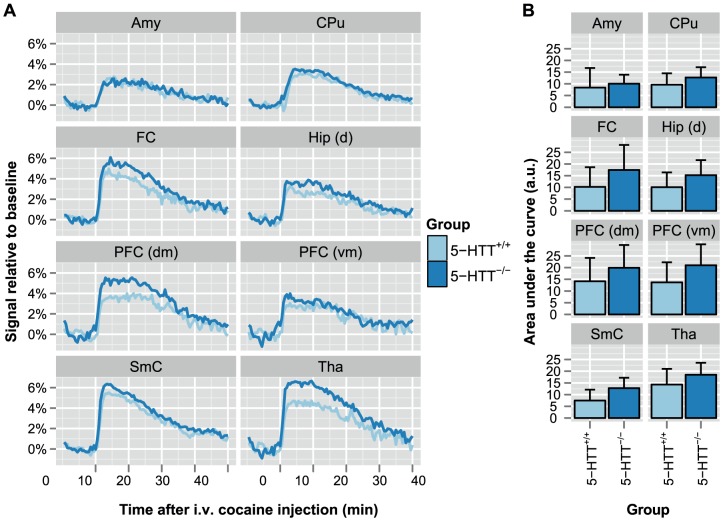
Cocaine-induced activation responses from seed-based analysis of pharmacological MRI. BOLD-based pharmacological MRI was performed for 50 minutes, with 1 mg/kg cocaine injected intravenously after 10 minutes. (A) The raw signal time-courses after preprocessing, and (B) the area-under-the curve of gamma-variate functions obtained with a Bayesian fitting routine, are displayed for a selection of seed regions. *Amy*: basal amygdaloid nuclei; *CPu*: caudate-putamen; *FC*: frontal cortex; *Hip (d)*: dorsal hippocampus; *PFC (dm)*: dorsomedial prefrontal cortex; *PFC (vm)*: ventromedial prefrontal cortex; *SmC*: somatosensory cortex; *Tha*: thalamic nuclei.

On average, the 5-HTT^-/-^ animals displayed a slightly higher pharmacological MRI profile following intravenous cocaine injection (Figure 4A). To test whether the group-mean responses were statistically different when accounting for confounding effects of physiological status on the BOLD response, we applied a generalized least squares fit with a correction for temporal autocorrelation, and included P_a_CO_2_ and MABP measures as covariates. We found significant (*p* < 0.05, uncorrected) group differences with a seed-based generalized least squares fit in the basal amygdaloid nuclei, dorsal raphe nuclei, frontal cortex, substantia nigra, temporal cortex, and visual cortex (Table 1). However, following correction for multiple comparisons, the FDR-adjusted *p*-values were all larger than 0.05.

**Table 1:** Cocaine-induced brain activation: results from seed-based generalized least squares fit of pharmacological MRI response.

Region	Coefficient ± SE	*t*-value	*p*-value	*d*
Dorsal hippocampus	–5.71×10^-2^ ± 9.19×10^-2^	–0.62	0.535	–0.03
Auditory cortex	–2.56×10^-1^ ± 1.35×10^-1^	–1.90	0.057	–0.08
Basal amygdaloid nuclei	1.22×10^-1^ ± 5.7×10^-2^	2.13	0.033*	0.09
Caudate-putamen	–4.56×10^-2^ ± 9.84×10^-2^	–0.46	0.643	–0.02
Dorsomedial prefrontal cortex	–2.18×10^-1^ ± 1.54×10^-1^	–1.42	0.156	–0.06
Dorsal orbitofrontal cortex	–8.15×10^-3^ ± 1.63×10^-1^	–0.05	0.960	–0.00
Dorsal raphe nuclei	–1.96×10^-1^ ± 7.92×10^-2^	–2.47	0.014*	–0.12
Frontal cortex	–2.33×10^-1^ ± 1.13×10^-1^	–2.06	0.040*	–0.09
Globus pallidus	–5.12×10^-3^ ± 3.94×10^-2^	–0.13	0.897	–0.01
Insula	–5.61×10^-2^ ± 1.5×10^-1^	–0.38	0.708	–0.02
Motor cortex	–1.46×10^-1^ ± 1.62×10^-1^	–0.90	0.366	–0.04
Nucleus accumbens	–3.77×10^-2^ ± 9.31×10^-2^	–0.41	0.685	–0.02
Parietal cortex	–9.71×10^-2^ ± 1.59×10^-1^	–0.61	0.542	–0.03
Ventral hippocampus	–1.3×10^-1^ ± 9.03×10^-2^	–1.44	0.150	–0.06
Retrosplenial cortex	–1.97×10^-1^ ± 1.57×10^-1^	–1.26	0.208	–0.05
Substantia nigra	–3.11×10^-1^ ± 1.03×10^-1^	–3.02	0.003**	–0.13
Somatosensory cortex	–1.11×10^-2^ ± 1.71×10^-1^	–0.07	0.948	–0.00
Temporal cortex	–4.34×10^-1^ ± 9.38×10^-2^	–4.63	0.000**	–0.20
Thalamic nuclei	–2.02×10^-1^ ± 1.24×10^-1^	–1.63	0.104	–0.07
Visual cortex	–3.47×10^-1^ ± 1.67×10^-1^	–2.08	0.037*	–0.09
Ventromedial prefrontal cortex	8.72×10^-2^ ± 1.71×10^-1^	0.51	0.610	0.02

Coefficients, standard error (SE), *t*-value, *p*-value, and effect size (Cohen’s *d*), for generalized least squares analysis of differences in the pharmacological MRI response between 5-HTT^+/+^ and 5-HTT^-/-^ rats. Response and predictor variables were normalized. *p < 0.05, **p < 0.005, uncorrected.

Using a hierarchical Bayesian approach, we fitted a three-parameter gamma-variate function on the average time signal in each ROI, for each subject separately. From the fits we calculated the estimated peak amplitude (maximum relative BOLD response), as well as the area under the curve. Linear mixed-model analyses found no significant overall differences between the two groups for maximum response and area under the curve. We tested also with a generalized least squares regression for differences between the groups separately for each region of interest (Table 2). Differences for the area under the curve in the retrosplenial cortex, somatosensory cortex, and visual cortex (Figure 4B, Table 2) were only significant (*p* < 0.05) without correction for multiple comparisons.

**Table 2:** Cocaine-induced brain activation: results from Bayesian fit of gamma-variate function to pharmacological MRI data.

	Maximum of the BOLD response, relative to baseline (%)				Area under the curve (a.u.)			
Region	5-HTT^+/+^	5-HTT^-/-^	*t*	*d*	5-HTT^+/+^	5-HTT^-/-^	*t*	*d*
Dorsal hippocampus	3.3 ± 1.7	4.5 ± 2.0	0.85	0.33	10.1 ± 6.3	15.2 ± 6.5	1.09	0.42
Auditory cortex	8.3 ± 5.1	9.2 ± 3.3	0.02	0.01	18.5 ± 8.4	23.9 ± 7.2	1.15	0.45
Basal amygdaloid nuclei	2.8 ± 1.9	3.1 ± 1.2	–0.23	–0.09	8.4 ± 8.4	10.0 ± 3.9	–0.13	–0.05
Caudate-putamen	3.2 ± 1.6	3.8 ± 1.3	0.56	0.22	9.6 ± 4.9	12.7 ± 4.4	0.91	0.35
Dorsomedial prefrontal cortex	4.6 ± 2.9	6.0 ± 2.6	1.02	0.39	14.1 ± 10.0	19.9 ± 9.7	0.99	0.38
Dorsal orbitofrontal cortex	4.6 ± 2.9	3.8 ± 2.8	–0.65	–0.25	14.0 ± 9.5	12.3 ± 9.7	–0.52	–0.20
Frontal cortex	4.1 ± 3.6	6.1 ± 3.4	0.20	0.08	10.2 ± 8.4	17.5 ± 10.7	0.74	0.29
Globus pallidus	5.6 ± 2.8	5.2 ± 2.1	0.02	0.01	16.1 ± 8.2	16.0 ± 6.6	0.16	0.06
Insula	2.8 ± 1.8	3.7 ± 1.2	0.62	0.24	7.8 ± 5.7	10.8 ± 3.7	0.54	0.21
Motor cortex	4.6 ± 2.3	5.8 ± 1.8	0.93	0.36	13.6 ± 6.9	18.6 ± 6.1	1.27	0.49
Nucleus accumbens	4.6 ± 2.2	5.7 ± 1.7	1.23	0.48	13.4 ± 6.3	18.6 ± 4.9	1.82	0.71
Parietal cortex	2.5 ± 1.5	2.4 ± 1.9	–1.10	–0.43	6.2 ± 3.6	7.7 ± 6.5	–0.15	–0.06
Ventral hippocampus	7.2 ± 3.7	7.5 ± 2.7	–0.24	–0.09	18.0 ± 8.6	22.0 ± 7.5	0.53	0.20
Retrosplenial cortex	2.6 ± 1.5	4.0 ± 1.6	1.57	0.61	7.4 ± 4.6	12.9 ± 4.7	2.10*	0.81
Substantia nigra	5.0 ± 3.3	6.5 ± 2.1	0.79	0.31	11.9 ± 7.4	19.4 ± 7.5	1.65	0.64
Somatosensory cortex	3.1 ± 1.5	4.2 ± 1.4	1.17	0.45	7.5 ± 4.7	12.8 ± 4.4	2.22*	0.86
Thalamic nuclei	5.1 ± 2.4	6.1 ± 2.0	0.64	0.25	14.3 ± 6.7	18.5 ± 5.1	1.02	0.40
Visual cortex	5.0 ± 2.8	7.5 ± 2.7	1.63	0.63	12.8 ± 6.2	22.1 ± 7.2	2.56*	0.99
Ventromedial prefrontal cortex	4.5 ± 2.3	6.6 ± 2.9	1.20	0.46	13.7 ± 8.5	21.0 ± 8.9	1.14	0.44

Group-wise mean ± standard deviation, *t*-value from two-sample *t*-test, and effect size (Cohen’s *d*), for two parameters calculated from Bayesian fits of a gamma-variate function to the pharmacological MRI response after intravenous cocaine injection. *p < 0.05, uncorrected.

MCMC-sampled differences (5-HTT^+/+^ – 5-HTT^-/-^) between estimates of the group mean of gamma-variate function parameters were not significantly different from zero, i.e. with zero contained in the 95% credible interval (_95%_CI) around the mean, for shape (mean 0.20, _95%_CI  =  [-0.02, 0.42]), rate (mean 0.008, _95%_CI  =  [-0.010, 0.024]), and scale factor (mean -2.8, _95%_CI  =  [-11.3, 5.0]).

**Figure 5:**
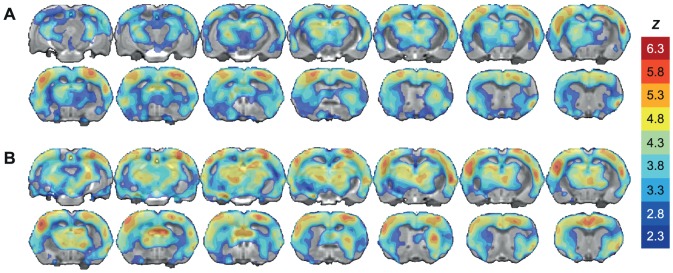
Cocaine-induced activation pattern from voxel-based analysis of pharmacological MRI. BOLD-based pharmacological MRI was performed for 50 minutes, with 1 mg/kg cocaine injected intravenously after 10 minutes. The maps display the group-level results from a voxel-based analysis of cocaine-induced brain activation, obtained from second-level analysis of subject-level generalized least squares fits of the pharmacological MRI signals in (A) 5-HTT^+/+^, and (B) 5-HTT^-/-^ animals. The maps display *Z*-values of significantly activated voxels (*p* < 0.01, cluster-corrected). Positive responses are color-coded between *Z*  =  [2.3, 6.3], and overlaid on a multi-slice anatomical rat brain template.

In a voxel-based analysis we observed widespread cocaine-induced activation in large parts of the cerebral cortex, as well as in hippocampus, dorsal and ventral striatum, and thalamic nuclei. Group mean response maps were calculated from a voxel-wise one-sample *t*-test (Figure 5). The cerebral pharmacological MRI response was found to be positive everywhere, and highly similar between wild-type and knockout rats. No significant differences between the two groups were found from a voxel-by-voxel fit.

### Diffusion tensor imaging

**Figure 6:**
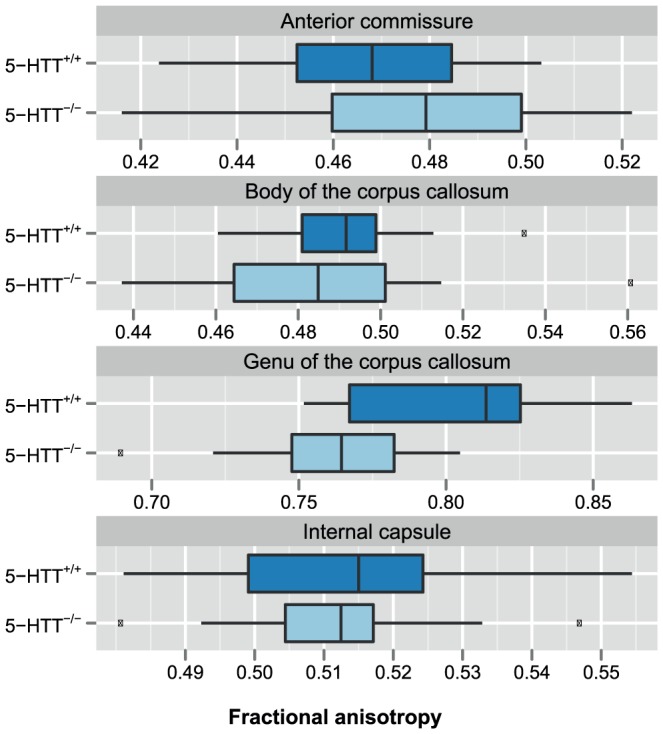
White matter fractional anisotropy values from seed-based analysis of fractional anisotropy maps. DTI fractional anisotropy was measured in four different white matter seed regions. Boxplots display for 5-HTT^-/-^ and 5-HTT^+/+^ animals the average value in each of the regions. Fractional anisotropy was significantly lower in the genu of the corpus callosum of 5-HTT^-/-^ animals (two-sample *t*-test, *t*  =  -3.33, false discovery rate (FDR)-adjusted *p* < 0.05).

At the voxel level, we found no evidence for differences in white matter integrity, as expressed by DTI fractional anisotropy values, between 5-HTT^+/+^ and 5-HTT^-/-^, using the TBSS approach. However, two-sample *t*-test showed that there was a significantly lower fractional anisotropy in the genu of the corpus callosum of 5-HTT^-/-^ animals as compared to wild-types (*t*  =  -3.33; FDR-adjusted *p* < 0.05; effect size, Cohen’s *d*  =  -1.31) (Figure 6). We did not find statistically significant differences in the other three white matter ROIs (anterior commissure: *d*  =  0.35; body of the corpus callosum: *d*  =  -0.31; internal capsule: *d*  =  -0.08). Differences in fractional anisotropy and mean diffusivity in gray matter regions also did not reach statistical significance (data not shown).

**Figure 7:**
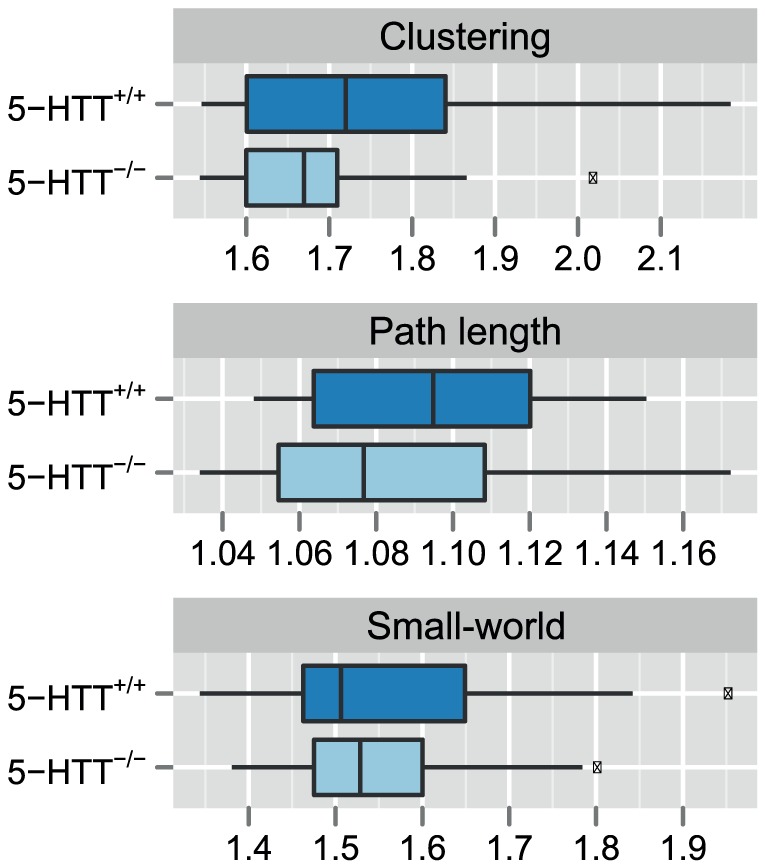
Network parameters from seed-based graph analysis of DTI tractography data. Structural brain networks were constructed from DTI-based fiber tracts among 23 bilaterally positioned cortical and subcortical gray matter ROIs. The edges between the regions were weighted by the mean fractional anisotropy along the tracts. The boxplots display three global network parameters that capture key properties of the networks: the clustering coefficient (i.e., average of local clustering coefficients), the characteristic path length (i.e., average of shortest path lengths), and the small-worldness (i.e., ratio of normalized clustering coefficient and normalized characteristic path length).

Similar to the functional brain network analysis, we obtained structural brain networks constructed from whole-brain DTI-based tractography and weighted by the average fractional anisotropy value along the connecting tracts. The normalized clustering coefficient was found to be 1.74 ± 0.19 in 5-HTT^+/+^ and 1.68 ± 0.13 in 5-HTT^-/-^ (effect size, Cohen’s *d*  =  -1.36), the normalized characteristic path length 1.10 ± 0.03 in 5-HTT^+/+^ and 1.09 ± 0.05 in 5-HTT^-/-^ (*d*  =  -0.90), and small-worldness 1.59 ± 0.18 in 5-HTT^+/+^ and 1.55 ± 0.12 in 5-HTT^-/-^ (*d*  =  -0.96) (Figure 7). Again, the ratio between normalized clustering coefficient and normalized characteristic path length indicated that the structural brain networks exhibit small-world characteristics. However, we found no significant differences in these global tractography-based graph parameters between wild-types and knockouts.

## Discussion

The 5-HTT^-/-^ phenotype has been associated with cocaine hyperresponsivity [10], increased stress sensitivity [7] and anxiety- and depression-like behavior [16] in rats. As reduced functional 5-HTT expression and subsequent increases in 5-HT levels may affect normal development of interconnections among corticolimbic structures implicated in anxiety-like traits [8], we applied various MRI paradigms to assess the effect of 5-HTT^-/-^ rat genotype on the functional and structural properties of neural networks under baseline conditions, and upon a rewarding cocaine stimulus. We observed typical functional connectivity patterns and robust hemodynamic responses following an intravenous cocaine injection, but our results do not suggest major alterations in baseline functional connectivity and cocaine-induced neural activity within the corticolimbic network. Similarly, white matter structural integrity seems largely unaffected by the lack of 5-HTT expression, except for the genu of the corpus callosum. At the macroscopic level, tractography-based graph analysis revealed that key properties of brain network organization were essentially similar between 5-HTT^-/-^ and 5-HTT^+/+^ rats.

The distinctive patterns of positive and negative functional connectivity, as displayed in seed-based resting-state fMRI functional connectivity maps, suggest broad segregation of functional networks. For instance, resting-state activity in the thalamic nuclei mostly correlated with that in retrosplenial and medial prefrontal regions, but not with that in caudate-putamen and somatosensory areas. However, cautious interpretation of negative and near-zero correlation values is warranted, as these may partly be the by-product of removing the global mean signal during pre-processing [26].

In 5-HTT^-/-^ mice, increased stress- and depression-like [19], [47] and abnormal anxiety-related behavior [48], [49] have been associated with morphological changes in ventromedial prefrontal cortex and basolateral amygdala [19], potentially contributing to changes in corticolimbic function. Spatiotemporal differences in neuronal tracer accumulation measured with manganese-enhanced MRI provided evidence for developmental alterations in the reward circuitry of 5-HTT^-/-^ mice, in the absence of significant differences in brain metabolite levels, gray matter morphology, and white matter integrity [21]. Here, we did not find significant differences between 5-HTT^+/+^ and 5-HTT^-/-^ rats with regard to resting-state fMRI functional connectivity values between specific ROIs, after correction for multiple comparisons. Furthermore, comparisons of functional neural networks using complex graph analysis parameters did not reveal major differences in key organizational properties between wild-types and knockouts. However, resting-state fMRI and manganese-enhanced MRI provide qualitatively different views of functional connections among the brain regions involved in reward processing. Whereas the former measures the instantaneous synchronicity of spontaneous neuronal signaling, the latter highlights direct anatomical pathways between neuronal populations. Although the uptake and trans-synaptic transfer of manganese is greatly facilitated by voltage-gated calcium channels [50], [51], anterograde axonal transport is not necessarily activity-dependent [52], [53]. The neural tracts thus traced provide an integrative measure of local structural connectivity and relative neuronal activity during the period after tracer injection. While resting-state fMRI-based functional connectivity is largely associated with direct structural connectivity (e.g., as measured with DTI), synchronization through indirect synaptic connections also contributes to the observed functional connectivity [54], [55]. Manganese-enhanced MRI of 5-HTT^-/-^ mice identified that the balance between mesolimbic, mesocortical, and nigrostriatal connections and activity was shifted towards the latter two pathways [21]. Our resting-state fMRI analysis did not reveal a difference in functional connectivity among the regions involved in these pathways, suggesting that the alterations in reward circuitry are not explained by changes in oscillatory coupling between these regions.

In human s-allele carriers, increased fMRI activation upon fearful stimuli [56], [57], and increased functional coupling between ventromedial prefrontal cortex and amygdala, possibly limiting the capacity to control emotional states under stressful conditions [58], has been explained by a reduced amygdala activation in the neutral versus fixation condition [59]. Resting-state quantitative EEG measurements linked low 5-HTT expression to overall lower spectral powers [60], and higher resting-state cerebral blood flow in amygdala and hippocampus, further supporting the hypothesis that 5-HTT genotype modulates resting activity rather than stimulus-induced activation [61]. In contrast, a recent study that addressed earlier reports of resting-state cerebral blood flow modulation in amygdala and ventromedial prefrontal cortex by 5-HTTLPR [62], concluded from a larger cohort that there was no association between 5-HTT genotype and baseline brain perfusion [63]. This is in agreement with our results, which indicate that baseline neural network configurations as measured with resting-state fMRI functional connectivity are generally independent of 5-HTT genotype.

5-HTT genotype has been associated with increased sensitivity to rewarding environmental stimuli like drugs of abuse, e.g. with s-allele carriers exhibiting increased alcohol use [64], [65] and drug dependence [65], and increased risk for developing cocaine use mediated by early environmental factors [66]. Similarly, 5-HTT^-/-^ mice and rats display increased cocaine-induced locomotor activity, cocaine-induced conditioned place preference, and intravenous cocaine self-administration [9]–[13]. On average, we found that 5-HTT^-/-^ rats displayed a slightly higher pharmacological MRI profile following intravenous cocaine, but after accounting for confounding differences in physiological status, and correcting for multiple statistical comparisons, the hemodynamic responses did not reflect the increased responsivity to cocaine as established in 5-HTT^-/-^ rats at the behavioral level [9], [10]. One important difference with human fMRI studies, and previous behavioral assessments in rats, is the necessary application of general anesthesia during pharmacological MRI. While cocaine inhibits ligand binding at the dopamine, serotonin and norepinephrine transporters [67], isoflurane further increases striatal dopamine levels [68] by reducing functional dopamine transporter availability [69]. Volatile anesthetics modulate serotonergic neurotransmission as well, with isoflurane decreasing extracellular 5-HT in mouse hippocampus [70] despite reduced synaptosomal uptake of 5-HT *in vitro* [71]. However, a stronger reduction of hippocampal 5-HT reuptake in 5-HTT^-/-^ as compared to 5-HTT^+/+^ mice [70] suggests that 5-HTT is not a major target of isoflurane *in vivo* in the present study. Furthermore, it has been shown that isoflurane and alpha-chloralose differentially affect baseline cerebral blood flow [72] and cocaine-induced hemodynamic and pharmacokinetic responses [73]. Although in the current study animals were minimally anesthetized with 1.0% isoflurane, drug-anesthetic interaction effects on cerebral hemodynamics and neurotransmitter function might in part explain differences between observations in awake and anesthetized animals, and potentially obscure the effects of 5-HTT genotype on BOLD-based measures of neural activity.

The potential effects of developmental alterations in 5-HT homeostasis are not limited to gray matter. It has been shown that axonal uptake of 5-HT is reduced by selective inhibition of 5-HTT [74]. Furthermore, the importance of early-life 5-HT homeostasis has been demonstrated by transient inhibition of 5-HTT function with selective serotonin reuptake inhibitors, which altered cortical network function, callosal axon myelination, and oligodendrocyte soma morphology in rat pups after perinatal citalopram treatment [75], while early postnatal fluoxetine treatment in mice produced similar deficits in emotional behavior as observed in 5-HTT knockouts [76]. Our seed-based DTI-analysis highlights a small but significant effect of 5-HTT genotype on fractional anisotropy values in the genu of the corpus callosum. This is consistent with the observation of lower fractional anisotropy values in the rostral body and genu of the corpus callosum, but not in other parts of the corpus callosum, in cocaine-dependent subjects, which paralleled impaired impulse control and reduced discriminability [77]. Furthermore, reductions in frontal white matter structural integrity have been reported in relation to 5-HTTLPR. Low 5-HTT expression has been associated with reduced fractional anisotropy along the frontal part of the uncinate fasciculus [78], and individual trait anxiety scores correlated negatively with fractional anisotropy along the pathway between amygdala and ventromedial prefrontal cortex [79]. The fractional anisotropy values in the corpus callosum of 5-HTT^+/+^ rats are in agreement with estimates previously obtained in normally developing rats [80]. Lower fractional anisotropy in the genu of the corpus callosum might thus indicate that normal callosal axon myelination is disturbed in 5-HTT^-/-^ rats, which may affect information integration across hemispheres. However, in the absence of histological data of callosal myelination that could provide support for the reductions in fractional anisotropy that we observed in the genu of the corpus callosum, and considering the potential contribution of fractional anisotropy values anterior to the corpus callosum due to partial-volume effects, one should be careful with interpretation.

Complex graph analysis measures of structural connectivity were not significantly different between 5-HTT^-/-^ and 5-HTT^+/+^ rats. However, these measures critically depend on the quality of the tractography results from which the underlying networks were constructed. Considering the spatial resolution of whole-brain *in vivo* DTI, these connections are most likely to reflect shortest paths along major white matter tracts. Likewise, the estimates of fiber exit points into gray matter tissue are limited by partial volume effects along the borders between gray and white matter. In general, the sensitivity of DTI and BOLD-based fMRI measurements to detect genotype effects were dependent on the achieved contrasts and spatial resolutions. The BOLD-based pharmacological MRI contrast is small compared to the contrast afforded by exogenous agents, which could thus improve the detectability of small genotype effects [81]. Furthermore, the fMRI-signal is highly dependent on neurovascular coupling. Here, manganese-based tracing of neural connections could provide an alternative and independent mapping of functional connections [51]. Finally, DTI-based tractography could benefit from higher spatial resolutions achieved using *post mortem* MRI at ultra-high field strengths [82].

Except for a few species-specific effects of 5-HTT gene knockout and subtle differences that depend on the inbred mouse strain background, many reported (endo)phenotypes are highly similar for both 5-HTT^-/-^ mice and rats (reviewed in [7], [83]). Inbred 5-HTT knockout mice are generated using homologous recombination of embryonic stem cells [84], [85], which only very recently became feasible in rats [86]. In contrast, the outbred 5-HTT^-/-^ rat that was used in this study has been obtained through ENU-driven target-selected mutagenesis [87]. Whereas this approach may have left undetected mutations in other parts of the genome, the similarities with mouse knockouts suggests that the influence of any unknown additional mutation must be minimal. Complete absence of functional 5-HTT expression in 5-HTT^-/-^ rodents may be perceived as an extreme condition, considering that 5-HTT function in s-allele carriers of 5-HTTLPR is reduced but not absent [7], [88]. Nevertheless, convergence of endophenotype effects of 5-HTT knockout in rodents and the similarities with brain and behavioral phenotypes in human s-allele carriers of 5-HTTLPR suggests that the use of 5-HTT^-/-^ rodents may be helpful in understanding 5-HTT genotype effects on brain and behavior [8].

In the absence of functional 5-HTT expression in mice, functional and structural defects have been found in regions along pathways involved in somatosensory and visual processing [89]–[92]. It is tempting to speculate that the marginally significant increase in functional connectivity between the somatosensory cortex and insula contributes to the increased social cognition as proposed by Canli and Lesch [93], given that these regions are involved in interoceptive awareness and empathy [94]. Furthermore, the connectivity between the visual cortex, somatosensory cortex and ventromedial prefrontal cortex may serve as a pathway by which 5-HTT^-/-^ rodents and 5-HTTLPR s-allele carriers engage into hypervigilance [8]. However, when considering the limited group sizes of the current study while multiple statistical tests of pairwise functional connectivity values were applied, control of the false discovery rate is appropriate and precludes conclusive acknowledgement of the aforementioned differences in functional connectivity.

In sum, our extensive MRI analysis of brain morphology and function in 5-HTT^-/-^ versus wild-type rats reveals several new brain phenotypes. For instance, a decrease in fractional anisotropy in the genu of the corpus callosum was observed, which may imply that long-distance connectivity is altered in 5-HTT^-/-^ rats, with potential consequences for the integration of information across hemispheres. Although the precise morphological nature and the functional implications remain to be identified, such a large-scale effect may contribute to multiple phenotypes as observed in 5-HTT^-/-^ rats.
